# Cerebrospinal Fluid IgM Levels in Association With Inflammatory Pathways in Multiple Sclerosis Patients

**DOI:** 10.3389/fncel.2020.569827

**Published:** 2020-10-16

**Authors:** Roberta Magliozzi, Valentina Mazziotti, Luigi Montibeller, Anna I. Pisani, Damiano Marastoni, Agnese Tamanti, Stefania Rossi, Francesco Crescenzo, Massimiliano Calabrese

**Affiliations:** ^1^Neurology Section of Department of Neurological and Movement Sciences, University of Verona, Verona, Italy; ^2^Division of Brain Sciences, Department of Medicine, Imperial College London, London, United Kingdom; ^3^Department of Oncology and Molecular Medicine, National Institute of Health, Rome, Italy

**Keywords:** CSF, multiple sclerosis, B cell, lesion activity, immunoglobulin M

## Abstract

**Background:**

Intrathecal immunoglobulin M (IgM) synthesis has been demonstrated in the early disease stages of multiple sclerosis (MS) as a predictor factor of a worsening disease course. Similarly, increased cerebrospinal fluid (CSF) molecules related to B-cell intrathecal activity have been associated with a more severe MS progression. However, whether CSF levels of IgM are linked to specific inflammatory and clinical profile in MS patients at the time of diagnosis remains to be elucidated.

**Methods:**

Using customized Bio-Plex assay, the protein levels of IgG, IgA, IgM, and of 34 other inflammatory molecules, related to B-cell, T-cell, and monocyte/macrophage activity, were analyzed in the CSF of 103 newly diagnosed relapsing–remitting MS patients and 36 patients with other neurological disorders. CSF IgM levels were also correlated with clinical and neuroradiological measures [advanced 3-T magnetic resonance imaging (MRI) parameters], at diagnosis and after 2 years of follow-up.

**Results:**

A 45.6% increase in CSF IgM levels was found in MS patients compared to controls (*p* = 0.013). CSF IgM levels correlated with higher CSF levels of CXCL13 (*p* = 0.039), CCL21 (*p* = 0.023), interleukin 10 (IL-10) (*p* = 0.025), IL-12p70 (*p* = 0.020), CX3CL1 (*p* = 0.036), and CHI3L1 (*p* = 0.048) and were associated with earlier age of patients at diagnosis (*p* = 0.008), white matter lesion (WML) number (*p* = 0.039) and disease activity (*p* = 0.033) after 2 years of follow-up.

**Conclusion:**

IgMs are the immunoglobulins mostly expressed in the CSF of naive MS patients compared to other neurological conditions at the time of diagnosis. The association between increased CSF IgM levels and molecules related to both B-cell immunity (IL-10) and recruitment (CXCL13 and CCL21) and to macrophage/microglia activity (IL-12p70, CX3CL1, and CHI3L1) suggests possible correlation between humoral and innate intrathecal immunity in early disease stage. Furthermore, the association of IgM levels with WMLs and MS clinical and MRI activity after 2 years supports the idea of key role of IgM in the disease course.

## Introduction

Multiple sclerosis (MS) is a chronic inflammatory demyelinating and neurodegenerative disease that affects the central nervous system (CNS). The presence of oligoclonal bands (OCBs) in the cerebrospinal fluid (CSF), but not within the serum, is a strong indicator of intrathecal antibody synthesis (Ziemssen et al., 2019) and has been considered a hallmark of MS that contributes to the diagnosis (Thompson et al., 2018). Immunoglobulin G (IgG) and IgM antibodies are major contributors to the OCBs formation in the CSF (Ziemssen and Ziemssen, 2005; Ziemssen et al., 2019). Elevated intrathecal IgM synthesis, but not other immunoglobulins, has been suggested to predict a worse disease evolution since the early MS stages, being associated in patients with a more disability progression and with a more aggressive form of the disease (Walsh and Tourtellotte, 1986; Villar et al., 2002, 2003; Cepok et al., 2006; Durante et al., 2012; Beltrán et al., 2014; Frau et al., 2018). In patients with clinically isolated syndrome (CIS), IgM OCB detection was associated with higher risks of conversion to clinically definite MS and in relapsing–remitting (RRMS) patients predicted a higher probability of converting to secondary progressive MS (Villar et al., 2002). In addition, the presence of colocalizing immunoglobulins and complement depositions in ongoing MS lesions (Genain et al., 1999) and studies demonstrating that antibodies isolated from the CSF of MS patients induce axonal damage and complement-mediated demyelination when applied to human CNS tissue *ex vivo* or *in vitro* (Elliott et al., 2012; Blauth et al., 2015), strongly support a key role of plasma cells and immunoglobulins, in MS pathology. It is known that B cells play a pathogenic role in MS both through the production of antibodies in the CNS and the release of proinflammatory factors in the CSF (Li et al., 2018; Sabatino et al., 2019), such as cytokines [tumor necrosis factor (TNF), interferon IFN (IFNγ), interleukin 6 (IL-6), IL-10, IL-34, IL-35, and granulocyte–macrophage colony-stimulating factor (GM-CSF)] and lymphoid chemokines (CXCL10, CXCL12, CXCL13) (Gardner et al., 2013; Li et al., 2015; Magliozzi et al., 2018). The release of B cells–related factors in the CSF has been suggested to have a key role in intrathecal inflammation, which could be linked to neuronal loss and microglia/macrophage activation and a worse MS course (Magliozzi et al., 2007, 2010; Haider et al., 2016; Lisak et al., 2017; Touil et al., 2017). More recently, increased CSF expression of similar inflammatory pattern related to B-cell immunity and lymphoid neogenesis was found associated with increased cortical lesion (CL) load, as revealed by advanced 3-T double inversion recovery magnetic resonance imaging (MRI) analysis, also in MS patients at time of diagnosis (Magliozzi et al., 2018). These evidences support the hypothesis of complex intrathecal immune interactions and potential correlations between intrathecal antibody syntheses by plasma cells, B cells–related factors release innate immune activities, which occur since early disease stages (Magliozzi et al., 2019). However, the definite inflammatory CSF milieu associated with increased CSF IgM levels remains to be better clarified.

The objective of this study was to evaluate the presence of IgM, and others immunoglobulins (IgG and IgA) in the CSF of both MS patients and controls with other neurological disorders. Furthermore, we investigated the existence of specific correlations between CSF IgM levels and the CSF inflammatory profiling at the time of diagnosis and the clinical and MRI activity in addition to baseline, even after 2 years of follow-up in a cohort of MS patients.

## Materials and Methods

### Patients Cohort

We recruited 103 treatment-naive RRMS patients (26 males and 77 females), followed at the MS Centre at Verona University Hospital (Italy), who received a diagnosis of MS from January 2012 to December 2019. All MS patients underwent at the time of diagnosis (T0) a detailed neurological evaluation including the Expanded Disability Status Scale (EDSS) assessment (Kurtzke, 1983) and the 3-T MRI and the CSF examination. Seventy patients who did not undergo second-line therapies, in particular anti–B-cell drug treatments, were also monitored from a clinical and radiological point of view for 2 years [24 months (T24)]. The evidence (EDA) and no evidence (NEDA) of disease activity, based on the presence of relapses and/or disability progression and/or any MRI activity (Giovannoni et al., 2015), were evaluated.

Demographic, clinical, and MRI data of MS patients are reported in Table 1.

**TABLE 1:** Demographic, clinical, and MRI data of MS patients.

Gender (male/female): T0, T24	26/77, 17/53
Age at diagnosis (years)	38.6 ± 13.2, 15–64
EDSS-T0	2.0 ± 2.0, 0–5
EDSS increase-T24	2.0 ± 0.0, 0–5
OCBs (positive/negative)	75/28
Albumin CSF/serum (mg/L)	4.6 ± 0.2, 2–11
WMLs number-T0	7.0 ± 2.0, 3–18
CL number-T0	2.0 ± 7.0, 0–23
New WMLs-T24	70
New CLs-T24	73
Relapses T0-T24	68
EDA/NEDA-T24	40/30

*M, males; F, females; EDSS, Expanded Disability Status Scale; OCBs, oligoclonal bands; WMLs, white matter lesions; CLs, cortical lesions; EDA, evidence of disease activity; NEDA, no evidence of disease activity; T, timepoint (0 = baseline, 24 = after 2 years of follow-up). Data are shown in the following order: mean ± SD (standard deviation) and range, except for WMLs, CLs, and EDSS, for which the median ± IQR (interquartile range) are considered in place of mean and SD.*

Thirty-six age- and sex-matched available patients affected by other neurological diseases, who underwent neurological evaluation and CSF examination at the time of the diagnosis, were included in the study. This group included 21 individuals with non-inflammatory neurological diseases, NIND (one idiopathic tremor, two migraine, two amyloid angiopathy, two fibromyalgia, four ischemic stroke, one spondylotic myelopathy, two amyotrophic lateral sclerosis, one olivopontocerebellar atrophy, one idiopathic spastic paraparesis, one idiopathic ataxia, one myopathy, one endocranial hypertension, two peripheral neuropathy), and 15 subjects with other inflammatory neurological diseases, OIND (one infective myelopathy, two CNS lymphoma, two intracranial abscess, one peripheral neuropathy, two Behçet disease, three neuromyelitis optica spectrum disorder, three autoimmune encephalitis, one aseptic meningitis).

The Ethics Committee of the University of Verona approved the study, and informed consent was obtained from all participants.

### CSF Collection and Analysis

CSF samples were obtained at least 1 month after the last relapse and within 2 months from the MRI acquisition, according to the Consensus Guidelines for CSF and Blood Biobanking (Teunissen et al., 2009). Once collected, the CSF was centrifuged, and the supernatant was divided from the cell pellet and stored at −80°C until use (Ethics Committee Protocol n° 66418). OCB presence was evaluated by isoelectric focusing on agarose gel for each MS patient group, as reported in Table 1. Protein presence and levels in the CSF were assessed by multiplex technology using a Bioplex assay (Bio-Plex X200 System equipped with a magnetic workstation, BioRad, Hercules, CA, United States) as previously described (Magliozzi et al., 2018). CSF samples were diluted 1:2 in phosphate-buffered saline to reach the optimal concentration. The immunoglobulin protein analysis (IgG1, IgA, IgM) was performed by using a custom Bio-Plex Pro-Human Isotyping Panel (3-plex). In addition, levels of specific inflammatory chemokines and cytokines associated with several pathways were evaluated including B-cell pathway (APRIL, LIGHT, TWEAK, BAFF, CXCL12, CXCL13, CCL21, IL-10, IL-34, IL-35, and GM-CSF), T-cell pathway (IFNγ, IFNα2, IL-4, IL-8, IL-22, CCL19, CCL20, and CCL25), monocyte/macrophage pathway (IL-1β, IL-6, CCL2, CCL8, CX3CL1, CXCL10, CXCL11, CHI3L1, sCD163, MMP1, and MMP2), and TNF pathway (TNFα, sTNFR1, and sTNFR2), as previously optimized (Magliozzi et al., 2018). These analytes were assayed by using customized kits Bio-Plex Pro Human Chemokine Panel-40-Plex and Bio-Plex Pro Human Inflammation Panel 1-37-Plex as previously described (Magliozzi et al., 2018) (see Supplementary Material). All samples were run in duplicate in the same experiment and in two consecutive experiments in order to verify the reproducibility and consistency of the results. The CSF analysis was performed by two independent investigators (RM and SR), blinded with respect to the clinical/radiological characteristics of each patient. Index of blood–brain barrier damage was calculated considering the CSF/serum albumin ratio and used to verify the potential correlation between IgM intrathecal synthesis and peripheral one.

### MRI Acquisition Protocol and Analysis

At the time of diagnosis, 3-T MRI was performed in all MS patients, and this was repeated 2 years after diagnosis on 70 of these patients. MRI sequences were acquired at the Radiology Unit of the University Hospital of Borgo Trento (Verona, Italy) using a Philips Achieva 3T MR Scanner (Philips Medical Systems, Best, Netherlands) as previously described (Magliozzi et al., 2018). The following image sets were acquired:

• 3D T1-weighted turbo field echo [repetition time (TR)/echo time (TE) = 8.4/3.7 ms, voxel size of 1 mm × 1 mm × 1 mm), total acquisition time of 5:51 min;
• 3D double inversion recovery (DIR) (TR/TE = 5,500/292 ms, inversion times (TI) TI1/TI2 = 525/2,530 ms voxel size of 1 mm × 1 mm × 1 × mm), turbo spin echo (TSE) readout with an optimal variable flip angle scheme and number of excitations (3, with total acquisition time of 10:49 min;
• 3D fluid-attenuated inversion recovery (FLAIR) (TR/TE = 5,500/292 ms, TI = 1,650 ms voxel size of 1 mm × 1 mm × 1 mm), same TSE readout as the DIR sequence, number of excitations 1, with total acquisition time 5:44 min.

The number of T2 hyperintense white matter lesions (WMLs) and CLs were identified on FLAIR and DIR images by an observer with a large experience on MS. The number of CLs was assessed following the recommendations for CLs scoring in patients with MS (Geurts et al., 2011). Owing to the suboptimal performance of the image-acquisition sequences on MRI in visualizing subpial lesions, the present analysis has taken into account mainly the intracortical and leukocortical lesions. MRI data are reported in Table 1.

### Statistical Analysis

Mann–Whitney *U* test was used to test differences between MS patients and control group, as well as differences between MS patients stratified by CL number at diagnosis (</>4, where 4 was the mean of the CL number in all the examined patients) and by the presence or not of the EDA after 2 years of follow-up. Analysis of variance (ANOVA) followed by *post hoc* pairwise comparison using the Tukey test was used to evaluate difference among immunoglobulin CSF levels in MS patients.

Pairwise univariate Spearman rank index was used to evaluate the correlation between CSF IgM levels and demographic and clinical MRI parameters (both at diagnosis and after 2 years of follow-up) and several inflammatory/immune-mediated pathways. Differences between males and females and between those patients with or without OCBs were tested by Mann–Whitney *U* test. *p* < 0.05 was considered statistically significant. GraphPad (version 5.0) and R software (version 3.5.3) were used to perform the analysis.

## Results

### Immunoglobulin CSF Expression in MS Patients and Controls

Intrathecal levels of IgG, IgA, and IgM were investigated in the CSF of 103 RRMS patients and 36 controls with other neurological disorders (Table 2).

**TABLE 2:** Immunoglobulins assayed in the CSF of MS and control groups.

**Immunoglobulin type**	**MS patients (*n* = 103)**		**Controls (*n* = 36)**	
	**Mean ± SD (ng/mL)**	**Concentration range (ng/mL)**	**Mean ± SD (ng/mL)**	**Concentration range (ng/mL)**
IgG	3,778 ± 2,760	365–8705	3,927 ± 64,059	315–19,042
IgA	1,707 ± 3,522	0.0–7977	6,244 ± 18,929	60–82,928
IgM	1,991 ± 1,396.9	67–6114	1,367 ± 1,560	104–17,910

*Ig, immunoglobulin; SD, standard deviation. IgG, IgA, and IgM were analyzed by Bio-Plex Pro-Human Isotyping Panel (3-plex), from the CSF of MS patients (*n* = 103) and control cases (*n* = 36).*

**FIGURE 1:**
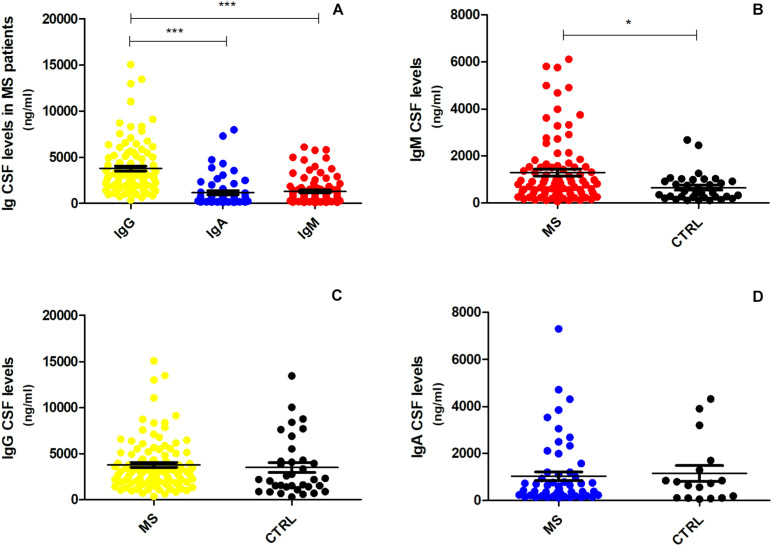
CSF expression levels of IgG, IgA, and IgM in MS and controls patients. **(A)** IgG levels were significantly higher in MS patients (*n* = 103) compared to IgA and IgM levels (one-way ANOVA, *p* < 0.001); **(B)** IgM was the only immunoglobulin found to be higher in MS patients (*n* = 103) compared to controls (*n* = 36) (Mann–Whitney *U* test, *p* = 0.013). The difference between **(C)** IgG and **(D)** IgA expression levels was not significant (respectively, *p* = 0.360, *p* = 0.700) between MS patients and controls. *p < 0.05, ***p < 0.001.

CSF IgG levels in MS patients were significantly higher compared to IgA (fold change = 2.65, *p* < 0.001; Figure 1A) and IgM (fold change = 2.15, *p* < 0.001; Figure 1A). However, only the CSF IgM levels reached statistical significance in MS patients when compared to controls (fold change = 1.46, *p* = 0.013; Figure 1B), while there was no difference in IgG (fold change = 0.96, *p* = 0.360; Figure 1C) and IgA (fold change = 0.27, *p* = 0.700; Figure 1D) levels between the two groups. In order to understand whether CSF IgM levels were related to peripheral ones and inflammation, we have further correlated CSF IgM concentration with measurement of blood–brain barrier damage (calculated considering the CSF–to–serum albumin ratio). However, we found no correlation (*R* = −0.12, *p* = 0.270) between CSF IgM levels and blood–brain barrier alteration, suggesting that IgM intrathecal levels were not related to peripheral ones.

### Correlation Between CSF IgM Levels With Demographic, Clinical, and MRI Data

CSF IgM levels of MS patients were correlated with demographic, clinical, and MRI data, at the time of diagnosis and after 2 years of follow-up. The results of these correlations were reported in Table 3.

**TABLE 3:** Correlation between CSF IgM levels with demographic, clinical, and MRI data.

	***R***	***p***
Gender (male/female)	—	0.313
**Age at diagnosis**	**−0.26**	**0.008**
EDSS T0	−0.04	0.678
EDSS change T24	0.09	0.498
OCBs	–	0.432
Albumin CSF/serum	−0.12	0.270
WMLs number –T0	0.03	0.740
CLs number-T0	0.15	0.130
**New WMLs-T24**	**0.24**	**0.039**
New CLs-T24	0.17	0.140
Relapses T0-T24	0.11	0.340
**EDA/NEDA**	**–**	**0.033**

*M, males; F, females; EDSS, expanded disability status scale; OCBs, oligoclonal bands; WMLs, white matter lesions; CLs, cortical lesions; EDA, evidence of disease activity; NEDA, no evidence of disease activity; T, timepoint (0 = baseline, 24 = after 2 years of follow-up); *R*, Spearman correlation coefficient. Spearman correlation coefficient (*R*) was used to evaluate the correlation between CSF levels of IgM levels with demographic, clinical, and MRI data in MS patients, except for differences between males and females, EDA and NEDA patients, and between those patients with or without OCBs that were tested by Mann–Whitney test. Demographic, clinical, and MRI data that correlated with IgM are reported in bold.*

**FIGURE 2:**
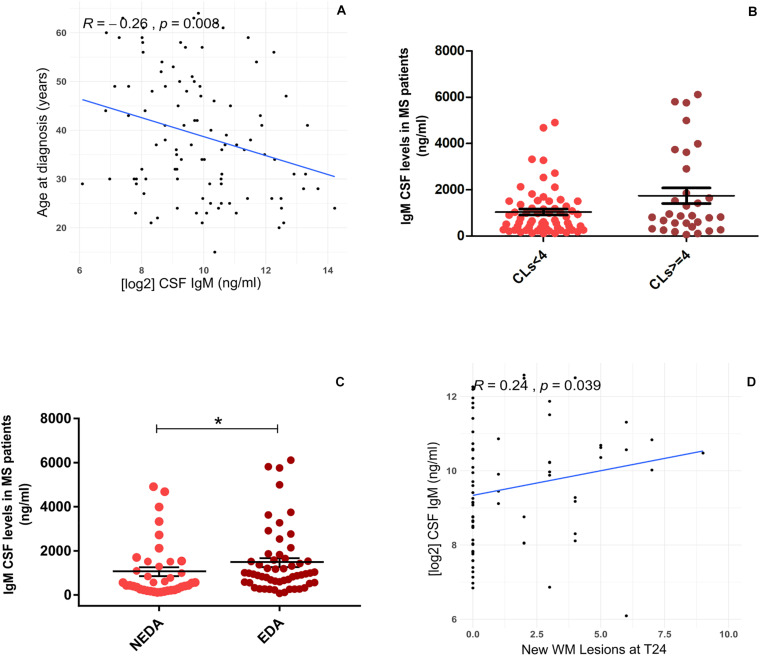
Association between CSF IgM levels in MS patients with demographic, clinical, and MRI data: **(A)** CSF IgM levels correlates negatively with the age at diagnosis (*R* = −0.26, *p* = 0.008); **(B)** CSF IgM levels were higher in MS patients with CLs ≥ 4 compared to MS patients with CLs < 4, at the time of diagnosis (Mann–Whitney *U* test, *p* = 0.072); **(C)** CSF IgM levels were higher in EDA patients compared to NEDA after 2 years of follow-up (*p* = 0.033); **(D)** CSF IgM levels correlate with the presence of new WMLs after 2 years of follow-up (*R* = 0.24, *p* = 0.039). *p < 0.05.

At T0, CSF IgM levels were correlated negatively with the age of MS patients (*R* = −0.26, *p* = 0.008; Figure 2A); on the contrary, no correlation was found between CSF IgM levels and EDSS and MRI data. Although there were no significant correlations between the CSF IgM levels and WMLs and CL number at diagnosis, when MS patients were stratified according to the CL load, we found a trend to increase of CSF IgM levels that were almost twice higher in MS patients with CLs ≥ 4 compared to MS patients with CLs < 4 (fold change = 1.74, *p* = 0.072; Figure 2B).

Considering the second year of follow-up, CSF IgM levels (measured at the time of diagnosis) were significantly higher in patients with EDA compared to NEDA (fold change = 1.61, *p* = 0.033; Figure 2C), and a mild correlation was found between CSF IgM levels and the presence of new WMLs (*R* = 0.24, *p* = 0.039; Figure 2D). By further analyzing the potential correlation between both IgG and IgA with all the tested clinical and radiological parameters, either at T0 and T24, we did not find any significant correlation.

### Correlation Between CSF IgM Levels and Inflammatory Profiles

**FIGURE 3:**
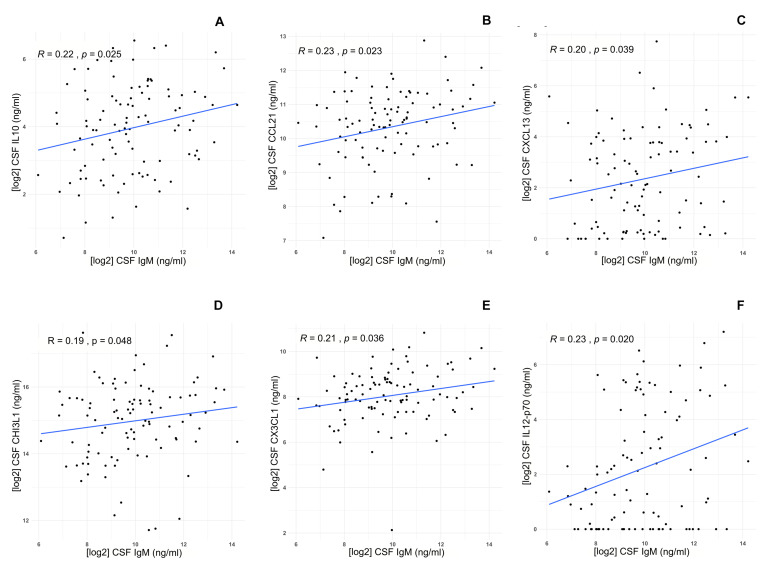
Correlations between IgM CSF levels and specific biomarkers in MS patients (*n* = 103). IgM CSF levels correlate with high CSF levels of **(A)** IL-10 (*R* = 0.22, *p* = 0.025), **(B)** CCL21 (*R* = 0.22, *p* = 0.023), **(C)** CXCL13 (*R* = 0.20, *p* = 0.039), **(D)** CHI3L1 (*R* = 0.19, *p* = 0.048), **(E)** CX3CL1 (*R* = 0.21, *p* = 0.036), **(F)** IL-12p70 (*p* = 0.020).

CSF IgM levels of MS patients were correlated with specific inflammatory/immune-mediated molecules, which were categorized in several pathways (Table 4). Among all the B cell–related molecules, we found that CSF IgM levels mildly correlated with CSF IL-10 (*R* = 0.22, *p* = 0.025; Figure 3A), CCL21 (*R* = 0.23, *p* = 0.023; Figure 3B), and CXCL13 (*R* = 0.20, *p* = 0.039; Figure 3C). Furthermore, the CSF IgM levels also weakly correlated with macrophage and microglia-related biomarkers such as CHI3L1 (*R* = 0.19, *p* = 0.048; Figure 3D), CX3CL1 (*R* = 0.21, *p* = 0.036; Figure 3E) and IL-12p70 (*R* = 0.25, *p* = 0.020; Figure 3F).

**TABLE 4:** Correlation between CSF IgM levels and Biomarkers in MS patients.

**Pathways**	**Biomarker**	***R***	***p***
B-cell pathway	APRIL	0.17	0.089
	LIGHT	−0.17	0.082
	TWEAK	0.05	0.593
	BAFF	0.13	0.210
	CXCL12	−0.01	0.899
	**CXCL13**	**0.20**	**0.039**
	**CCL21**	**0.23**	**0.023**
	**IL-10**	**0.22**	**0.025**
	IL-34	0.17	0.089
	IL-35	−0.11	0.269
	GM-CSF	0.17	0.084
T-cell pathway	IFNγ	0.13	0.194
	IFNα2	−0.03	0.759
	IL-4	−0.05	0.617
	IL-8	0.14	0.147
	IL-22	0.19	0.062
	CCL19	0.13	0.617
	CCL25	0.16	0.111
Monocyte/macrophage pathway	IL-1β	0.21	0.584
	IL-6	0.16	0.111
	CCL2	0.05	0.584
	CCL8	−0.04	0.668
	IL-12 (p40)	0.01	0.931
	**IL-12 (p70)**	**0.23**	**0.020**
	**CX3CL1**	**0.21**	**0.036**
	CXCL10	0.08	0.424
	CXCL11	0.16	0.112
	**CHI3L1**	**0.19**	**0.048**
	sCD163	0.12	0.239
	MMP1	−0.13	0.184
	MMP2	0.16	0.100
**TNF pathway**	TNFα	0.10	0.296
	sTNFR1	0.04	0.688
	sTNFR2	−0.06	0.546

*APRIL, a proliferation-inducing ligand; LIGHT, TNF superfamily member 14 (TNFSF14); TWEAK, TNF-related weak inducer of apoptosis; BAFF, B cell–activating factor; CXCL, “C-X-C” motif Ligand; CCL, “C-C” motif ligand; IL, interleukin; GM-CSF, granulocyte–macrophage colony-stimulating factor; IFN, Interferon; CHI3L1, chitinase 3-like 1; sCD163, soluble cluster of differentiation 163; MMP, matrix metallopeptidase; TNF, tumor necrosis factor; sTNFR, soluble tumor necrosis factor receptor; *R*, Spearman correlation coefficient. Spearman correlation coefficient (*R*) was used to evaluate the correlation between CSF levels of IgM and the other examined biomarkers (cytokines and chemokines) in MS patients. Molecules that correlated with IgM are reported in bold.*

## Discussion

In the last decade, several studies have demonstrated a relevant association between intrathecally produced IgM and a more severe MS course (Villar et al., 2005, 2008; Calabrese et al., 2012). However, other studies did not find an association between IgM and a more severe MS course (Schneider et al., 2007; Stauch et al., 2011). In our study, we aimed to define quantitatively the intrathecal IgM levels and its possible association with combined specific inflammatory profile in the CSF of MS patients.

We found that higher levels of IgM, but not IgG and IgA, were present already at diagnosis in the CSF of MS patients when compared with patients with other neurological diseases. This result corroborates the hypothesis that IgM production occurs from the early stages of MS. As known in MS, B cells migrate from the periphery into the meninges, CSF, and the CNS parenchyma (Levinson et al., 1983; Sandberg et al., 1986), where these cells showed a local activation and clonal expansion (Weber et al., 2010). Plasma blasts and plasma cells, maturing from these B cells, were capable to secrete oligoclonal antibodies in the CSF of MS patients (Weber et al., 2010). MS patients showed an intrathecal production of IgG, IgM, and IgA (Lolli et al., 1989); about 95% of the MS patients displayed IgG OCBs (Link, 1978; Villar et al., 2005), and around 40% also showed intrathecal IgM production (Villar et al., 2005), while CSF IgA synthesis was only occasionally observed (in 13% of cases) (Link and Müller, 1971; Leary et al., 2000). Therefore, IgG (predominantly IgG1), and IgM are considered the major contributors to the OCB formation in the CSF (Ziemssen and Ziemssen, 2005; Ziemssen et al., 2019).

Intrathecal IgM synthesis is involved in demyelination and axonal injury (Piddlesden et al., 1993; Villar et al., 2005), the main source of disability in MS patients (Mead et al., 2002). In particular, IgMs are the only immunoglobulins capable of recognizing myelin lipids, such as myelin oligodendrocyte glycoprotein, proteolipid protein, and myelin basic protein, in most of MS patients (Villar et al., 2005, 2008; Owens et al., 2009). In relation with these studies, we decided to analyze the association between the CSF IgM levels, detected at diagnosis (T0), and the clinical and MRI parameters after 2 years of follow-up [24 months (T24)]. We found that while CSF IgM levels did not correlate with any clinical/MRI parameters at time of diagnosis, they were correlated, even if moderately, with the presence of new WMLs at T24 and were significantly higher in EDA patients compared to NEDA, thus suggesting a possible prognostic role of IgM levels in terms of disease activity. These results are in line with previously mentioned studies (Villar et al., 2005, 2008; Owens et al., 2009) and with studies showing an association between IgM with a severe MS course, according to both clinical and MRI outcomes (Villar et al., 2003, 2005;Ozakbas et al., 2017).

Analyzing the correlations between CSF IgM levels and the clinical and MRI parameters even at the time of diagnosis, we detected only a negative and low correlation between CSF IgM levels with and the age of patients at diagnosis, although previous studies showed that IgM levels were strongly associated with a younger age at first clinical symptoms (Tintore et al., 2008; Huss et al., 2018; Pfuhl et al., 2019). Moreover, despite that CSF IgM levels were higher in MS patients with high (CLs ≥ 4) compared to MS patients with low (CLs < 4) CL load, such a difference did not reach the statistical significance. On the contrary, we detected low negative correlation with age of the patients. These results might be explained by the small number of examined patients and by the possibility that other underlying immunological mechanisms are involved in brain damage besides the immunoglobulin production (Lassmann, 2008).

Recent studies have shown that intrathecal IgM synthesis, mainly mediated by CD5^+^ B cells, contributes to B-cell activation and differentiation within the CNS (Villar et al., 2010). In particular, positive correlations between CSF inflammatory biomarkers, especially of humoral immunity, with MS severity support a pathogenic role of intrathecal inflammation, particularly linked to B-cell immunity, in CNS tissue destruction in MS patients (Milstein et al., 2019), causing a more severe and rapid disease course (Magliozzi et al., 2007; Howell et al., 2011). For all these reasons, we investigated whether IgM overexpression in the CSF of MS patients at diagnosis might be associated with a specific inflammatory intrathecal milieu, by analyzing other cytokine/chemokine CSF molecules related to either B cells or other immune cell pathways. First, we observed mild correlation between CSF IgM levels with some B cell–related factors, such as CXCL13, CCL21, and IL-10. The chemokines CXCL13 and CCL21 are particularly known to regulate B-cell migration into the CNS and to favor the intrathecal accumulation of B cells (Kowarik et al., 2012). In particular, CXCL13 has recently been suggested as a prognostic marker for CIS and MS (Brettschneider et al., 2010; Ferraro et al., 2015; Magliozzi et al., 2020) and seems to play a role in the formation of ectopic lymphoid tissues within the CNS in MS (Magliozzi et al., 2007). In addition, CSF CXCL13 levels were found incremented in MS patients (Khademi et al., 2011), in which they are correlated with a high number of CSF CD5^+^ B cells and with intrathecal IgM production (Krumbholz et al., 2006; Villar et al., 2010; Ferraro et al., 2015). Moreover, the correlation between CSF IgM levels and IL-10 supports the hypothesis that the B-cell activity could regulate activation of further immune reactions, even independently by immunoglobulin/complement–mediated response, since the earliest phase of the disease (Li et al., 2018). IL-10 is a potent immunomodulatory cytokine that can promote humoral immune responses by enhancing class II expression on B cells and inducing immunoglobulin production (Saxena et al., 2015). However, IL-10 could be also released, together with IL-1, IL-6, IL-15, and TNF, by activated macrophages, which can have a key role in B-cell activation. We also found that the CSF IgM levels in MS patients weakly correlated also with molecules related to monocyte/macrophage activity and response, such as CHI3L1, IL-12p70, and CX3CL1, suggesting a mild, but significant, association between humoral and innate immune inflammatory processes. CHI3L1, also named YKL-40, is a molecule released by activated macrophages and astrocytes, and it was proposed as a putative CSF biomarker of disease activity, indicating higher risk of conversion from CIS to MS (Comabella et al., 2010). IL-12 protein is composed by p35 and p40 subunits; when combined, these subunits form the bioactive IL-12p70, which is mainly produced by dendritic cells, macrophages, neutrophils, and probably by naive B cells. IL-12p70 is involved in the response to the antigen presentation, possibly with anti-inflammatory function (Gee et al., 2009). CX3CL1, also named fractalkine, is a chemokine mainly produced by neurons and can be soluble, as well as membrane-bound capable of attracting T cells, NK cells, and myeloid cells, including microglia (Hatori et al., 2002; Savarin-Vuaillat and Ransohoff, 2007). It has a key role in neuron–microglia cross-talk in physiology and aging, but the exact role of this chemokine in MS pathology still remains unclear, because CX3CL1 appeared to interfere with proinflammatory microglia activity, therefore with neuroprotective effect (Ransohoff and El Khoury, 2015). We previously demonstrated that the CSF levels of other specific markers of activated macrophages, such as soluble CD163, positively correlated with CSF levels of neurofilament, fibrinogen, and B cell–related molecules, such as CXCL13, CXCL12, IL-10, and BAFF (Magliozzi et al., 2019). Therefore, although the correlations that we identified in the CSF at time of diagnosis between all these mediators and IgM were modest and need to be validated in a larger and independent MS population, it might be hypothesized that IgM could possibly reflect the interactions between innate and adaptive humoral immune responses, as previously suggested (Boes, 2000; Villar et al., 2010).

This analysis was performed using a multiple, advanced immunoassay methodology (Bio-Plex), to obtain a simultaneous, sensitive, and reproducible evaluation of immunoglobulins and several other inflammatory mediators in the CSF of MS patients. Despite further studies are needed to confirm these preliminary results, the sensitivity and reproducibility of this easily performed procedure could allow extending such a detailed CSF proteomic analysis to the clinical practice.

This study is not without limitations. The low number of patients recruited, compared to the number of parameters analyzed, and the limited MS phenotypes suggest that these data need to be further validated, considering an independent validation cohort, and confirmed by other studies. Moreover, only 2 years of follow-up have been analyzed at the moment in order to select only MS patients treated by first-line therapies, in order to avoid further confounding factor of the correlation analysis. All together, these limitations suggest that this study needs to be confirmed by further analysis of a larger sample size and longer follow-up and paired serum samples.

## Conclusion

In this study, we used a comprehensive, advanced proteomic approach for quantitative and qualitative CSF protein analyses in combination with clinical and radiological assessment of MS patients, demonstrating that intrathecal IgM levels are increased in the CSF of treatment-naive MS patients compared to controls and that there is significant correlation between CSF IgM levels and further CSF molecules related to B-cell, macrophage, and microglia activity. Moreover, we found an association between CSF IgM production and the number of new WMLs and MRI activity after 2 years of follow-up, suggesting that CSF IgM levels might reflect the relationship between humoral and innate intrathecal immune response in MS and might represent an early biomarker of underling disease activity.

## Data Availability Statement

The raw data supporting the conclusions of this article will be made available by the authors, without undue reservation.

## Ethics Statement

The Ethics Committee of the University of Verona approved the study. The patients/participants provided their written informed consent to participate in this study. Biological material and associated data were obtained from MSBioB Biological bank – A.O.U.I., Verona (Protocol number 66418, 25/11/2019). Biological material was obtained from voluntary donors in compliance with the Legislative Decree 196/2003 “Personal Data Protection Code.”

## Author Contributions

RM and MC contributed to the conception and design of the work. RM, VM, LM, AP, DM, AT, and SR contributed to the acquisition, analysis, and interpretation of data for the work. RM, VM, LM, AP, DM, and FC contributed to the data interpretation. RM, VM, LM, AP, DM, AT, and MC contributed to writing the manuscript. RM, VM, DM, FC, AP, AT, and MC contributed to revise the manuscript. All authors provided approval for publication of the final content.

## Conflict of Interest

The authors declare that the research was conducted in the absence of any commercial or financial relationships that could be construed as a potential conflict of interest.
