# Supplementary material for: Cerebrospinal Fluid IgM Levels in Association With Inflammatory Pathways in Multiple Sclerosis Patients
Source: Front Cell Neurosci. 2020 Oct 16;14:569827. doi: 10.3389/fncel.2020.569827 (PMC7596330; doi:10.3389/fncel.2020.569827)
Supplement: Supplementary file 1 [file Table_1.DOCX]

| **Supplementary Table** | |  |  |
| --- | --- | --- | --- |
| ***Cytokines and chemokines detected in the CSF of the MS patients.*** | | | |
| **Pathway** | **Biomarker** | **Mean ± SD** | **Concentration range** |
|  |  | **(ng/ml)** | **(ng/ml)** |
| *B cells pathway* | |  |  |
|  | APRIL | 49405 ± 57885 | 883 - 376209 |
|  | LIGHT | 271 ± 408 | 0 - 1638 |
|  | TWEAK | 2072 ± 1938 | 62 - 12645 |
|  | BAFF | 10469 ± 7292 | 2433 - 45027 |
|  | CXCL12 | 2119 ± 2442 | 39 -17937 |
|  | CXCL13 | 11 ± 24 | 0 - 213 |
|  | CCL21 | 1653 ± 1150 | 134 - 7519 |
|  | IL-10 | 21 ± 19 | 1 - 92 |
|  | IL-34 | 451 ± 568 | 0 - 3272 |
|  | IL-35 | 279 ± 202 | 0 - 905 |
|  | GM-CSF | 477 ± 19 | 76 - 88 |
| *T cells pathway* | |  |  |
|  | IFNγ | 27 ± 0 | 5 - 5 |
|  | IFNα2 | 81 ± 0 | 5 - 16 |
|  | IL-4 | 52 ± 0 | 9 - 12 |
|  | IL-8 | 190 ± 2 | 32 - 43 |
|  | IL-12 (p40) | 14 ± 0 | 2 - 3 |
|  | IL-12 (p70) | 126 ± 0 | 30 - 34 |
|  | IL-22 | 355 ± 0 | 25 - 69 |
|  | CCL19 | 387 ± 1 | 108 - 110 |
|  | CCL25 | 378 ± 11 | 124 - 88 |
| *Monocyte/macrophage pathway* | |  |  |
|  | IL-1β | 3 ± 4 | 0 - 19 |
|  | IL-6 | 1033 ± 0 | 56 - 197 |
|  | CCL2 | 1398 ± 149 | 47 - 279 |
|  | CCL8 | 1398 ± 0 | 88 - 309 |
|  | CX3CL1 | 354.2 ± 282 | 3 - 1813 |
|  | CXCL10 | 780 ± 24 | 269 - 201 |
|  | CXCL11 | 63 ± 0 | 5 - 13 |
|  | CHI3L1 | 552545 ± 1097 | 54814 - 101565 |
|  | sCD163 | 332026 ± 7 | 35503 - 70335 |
|  | MMP1 | 1199 ± 6 | 194 - 289 |
|  | MMP2 | 4869 ± 1 | 648 - 1016 |
| *TNF pathway* | |  |  |
|  | TNFα | 38 ± 37 | 2 - 223 |
|  | sTNFR1 | 4205 ± 2603 | 325 - 13782 |
|  | sTNFR2 | 993 ± 824 | 0 - 3921 |

*Abbreviations: APRIL=a proliferation-inducing ligand; LIGHT=TNF superfamily member 14 (TNFSF14); TWEAK=TNF-related weak inducer of apoptosis; BAFF=B cell Activating Factor; CXCL-=“C-X-C” motif ligand; CCL=*“*C-C”* *ligand; IL=interleukin; IFN=interferon; GM-CSF=granulocyte-macrophage colony-stimulating factor; CHI3L1****=****Chitinase 3-Like 1; sCD163=soluble Cluster of Differentiation 163; MMP=matrix metallopeptidase; TNF=tumor necrosis factor; sTNFR=soluble tumour necrosis factor-receptor.*
